# Steroids in Immune Checkpoint Inhibitor Myocarditis

**DOI:** 10.1016/j.jaccao.2024.07.002

**Published:** 2024-08-06

**Authors:** Nicolas L. Palaskas, Bilal A. Siddiqui, Anita Deswal

**Affiliations:** a Department of Cardiology, Division of Internal Medicine, The University of Texas MD Anderson Cancer Center, Houston, Texas, USA; b Department of Genitourinary Medical Oncology, Division of Cancer Medicine, The University of Texas MD Anderson Cancer Center, Houston, Texas, USA

**Keywords:** immunotherapy, myocarditis, treatment

Immune checkpoint inhibitors (ICIs) have emerged as essential cancer treatments with an increasing expansion in their indications. Despite the cancer therapeutic success of ICIs, a wide array of toxicities can occur in any organ system of the body, referred to as immune-related adverse events (irAEs). These irAEs are thought to be secondary to the loss of self-tolerance leading to an autoimmune inflammatory response. Several cardiovascular irAEs have been described, including myocarditis and pericarditis.^1^ ICI myocarditis is uncommon with an incidence around 1% but has the highest reported irAE mortality, initially up to 50%.^2^^,^^3^ Given the high mortality, timely recognition and optimal treatment regimens are needed in addition to preventive therapies. At present, the mainstay of treatment for most irAEs, including ICI myocarditis, is steroids. The optimal dosing, duration, and timing of steroids recommended by expert consensus guidelines are based on limited evidence. This viewpoint discusses the evolving role of steroids in treating ICI myocarditis.

The emergence of irAEs from ICIs over the past 15 years has led to several medical professional societies publishing irAE treatment guidelines, largely based on expert consensus opinion without high level of evidence, and corticosteroids are the primary recommended treatment.^4^, ^5^, ^6^, ^7^ However, given their adverse effects, nonsteroidal agents targeting specific immune pathways are increasingly being used for the treatment of ICI myocarditis. There are 4 key components of steroid therapy: dose, timing of initiation, duration of treatment, and consideration of additional or alternate nonsteroidal therapy.

## Steroid Dosing

All guidelines for ICI myocarditis recommend initial high-dose corticosteroids, with the majority recommending pulse dose intravenous methylprednisolone at 500 to 1,000 mg daily (Table 1).^4^, ^5^, ^6^, ^7^ Only the American Society of Clinical Oncology (ASCO) clinical practice guidelines for the management of irAEs recommend steroid initiation with lower doses of either oral or intravenous prednisone at 1 to 2 mg/kg daily, with an increase to pulse dose steroids in patients without an “immediate response.”^5^ Although there is not a clear definition of a “response” to therapy, most consider a response to include clinical variables, such as hemodynamic instability, arrhythmias and/or electrocardiogram changes, and troponin levels. Furthermore, the level of troponin reduction that constitutes a response is unclear. Rather than a threshold level, evaluating daily trends that indicate a clear decrease vs an increase or stagnation is preferred. The use of high-dose steroids is largely based on 1 retrospective observational study of an international cohort of 126 patients with ICI myocarditis.^8^ This study found that the initial use of high-dose steroids (501-1,000 mg/d) was associated with fewer major adverse cardiovascular events (MACE) compared to intermediate-dose (60-500 mg/d) and low-dose (<60 mg/d) steroids (22%, 55%, and 62% respectively; *P* < 0.001).^8^ Additionally, the ASCO and the National Comprehensive Cancer Network (NCCN) guidelines recommend high-dose steroids only for those with severity grades ≥2 and ≥3, respectively.^5^^,^^7^ In contrast, the European Society of Cardiology (ESC) and the Society for Immunotherapy of Cancer (SITC) guidelines do not distinguish between severity grades of ICI myocarditis for the use or dose of steroids.^4^^,^^6^ It is important to recognize the severity spectrum of ICI myocarditis because those with asymptomatic lower-grade troponin elevation are diametrically different from those presenting with life-threatening arrhythmias. Changes in clinical practice patterns with increased baseline troponin assessments as recommended by the ESC guidelines may help to limit the treatment of asymptomatic troponin elevation and reserve the use of early and high-dose steroids for those with symptoms and/or signs of myocarditis. Our own work has shown that a subgroup of patients with a lower severity of myocarditis based on a lower degree of troponin elevation and endomyocardial biopsy with only mild inflammatory infiltrate but without myocyte necrosis did not have adverse cardiovascular events despite no treatment with steroids.^9^ Further research is needed to evaluate whether lower-severity ICI myocarditis can be treated with lower-dose steroids and which clinical factors could identify such patients.

**Table 1:** Immune Checkpoint Inhibitor Myocarditis Steroid Therapy Recommendations

	ESC Cardio-Oncology Guidelines	ASCO CPG irAE Guidelines	SITC irAE Guidelines	NCCN irAE Guidelines	Our Approach
Initial dose and severity	500-1,000 mg/d IV for patients with nonfulminant and fulminant myocarditis	1-2 mg/kg/d oral or IV prednisone; if no immediate response, then 1,000 mg IV methylprednisolone; only for those with ≥grade 2 severity	1,000 mg/d IV methylprednisolone	1,000 mg/d IV methylprednisolone; only for those with ≥grade 3 severity	1,000 mg/d IV methylprednisolone daily for 3 days if patients present with MACE. Consider lower-dose steroids for asymptomatic or mild troponin elevations without another obvious etiology.
Timing	Within 24 hours	Within 24 hours	As soon as possible	No recommendation	Rapid initiation within 24 hours for patients with MACE without another etiology on initial evaluation; perform full diagnostic work-up for asymptomatic, mild severity disease, or low suspicion, and initiate steroids if a diagnosis of myocarditis is confirmed.
Duration	Pulse dose minimum 3 days 17- to 20-week taper	1-2 mg/kg prednisone and if no immediate response, use 1,000 mg IV methylprednisolone 4- to 6-week taper	Pulse dose for 3 to 5 days 4- to 6-week taper	Pulse dose for 3 to 5 days 4- to 6-week taper	Pulse dose steroids for 3 days followed by 5-week taper. Continue taper and if clinical deterioration or persistent troponin elevation, then use nonsteroidal immunomodulator rather than delay steroid taper. 5-week steroid taper • 1,000 mg IV methylprednisolone daily for 3 days • 1 mg/kg IV methylprednisolone daily for 7 days • 0.5 mg/kg orally prednisone daily for 7 days • 0.33 mg/kg orally prednisone for 7 days • 10 mg orally prednisone for 7 days • 5 mg orally prednisone for 7 days
Nonsteroidal immunomodulator therapy	Hemodynamically unstable fulminant myocarditis or steroid refractory	No immediate response or in life-threatening cases	No improvement within 24 hours on steroids	No improvement within 24 hours on steroids	Early initiation for patients with MACE, clinical deterioration, or lack of consistent troponin reduction

ASCO = American Society of Clinical Oncology; CPG = Clinical Practice Guideline; ESC = European Society of Cardiology; irAE = immune-related adverse event; IV = intravenous; MACE = major adverse cardiovascular event; NCCN = National Comprehensive Cancer Network; SITC = Society for Immunotherapy of Cancer.

Another consideration in determining the initial dose of steroids is the presence of concomitant irAEs, particularly neuromuscular irAEs, including myositis and myasthenia gravis, which portend worse prognosis. The neurology literature regarding non–ICI-related myasthenia gravis cautions against high-dose steroids because they may precipitate respiratory decline requiring intubation.^10^ In patients with ICI myocarditis and myasthenia gravis overlap that present with life-threatening cardiac conditions, such as complete heart block, we still proceed with high-dose steroids, attempting to reverse the cardiovascular events while accepting the potential risk of requiring mechanical ventilation. Additionally, in a single-center study of patients with ICI myasthenia gravis, the use of first-line plasmapheresis or intravenous immunoglobin in addition to steroids was associated with improved symptoms compared to steroids alone (95% vs 63%; *P =* 0.011).^11^ However, every patient should be evaluated individually, assessing the risks and benefits of such treatment in a multidisciplinary approach involving the oncologist, neurologist, and cardiologist.

## Steroid Timing

All guidelines recommend early initiation of steroid therapy after patients present with ICI myocarditis.^4^, ^5^, ^6^, ^7^ Both the ESC and ASCO guidelines specify a time frame within 24 hours of presentation.^4^^,^^5^ This recommendation is again largely based on the previously mentioned retrospective cohort, which showed receipt of steroids within 24 hours of presentation was associated with fewer persistent elevations in troponin, defined as troponin remaining above 50% of peak level, compared to steroid initiations between 24 and 72 hours of presentation and >72 hours from presentation (32%, 67%, and 41%, respectively; *P =* 0.026).^8^ It should be noted that the endpoint evaluated for early initiation of steroids was based on troponin levels and not MACE.^8^ Therefore, timing can be driven by the clinical presentation. If patients present with MACE, such as ventricular tachycardia, complete heart block, or cardiogenic shock, not explained by other causes, then rapid initiation of steroids pending further diagnostic work-up (cardiac magnetic resonance or endomyocardial biopsy results) is warranted. However, if patients are completely asymptomatic and otherwise medically stable, then we may cautiously wait to establish a firm diagnosis of ICI myocarditis because we believe this may be prudent to avoid overtreatment. The increased use of high-sensitivity troponin assays in institutions transitioning from conventional troponin to high-sensitivity assays may lead to earlier treatment in asymptomatic patients while providers become familiar with typical trends in high-sensitivity troponin in their specific patient populations. The second aspect of timing is when to initiate the steroid taper. The current guidelines recommend tapering after 3 to 5 days of pulse dose steroids, but the SITC and NCCN guidelines have caveats of tapering when the “troponin normalizes” and when the “cardiac function returns to baseline,” respectively.^6^^,^^7^ In our experience, troponin levels, especially troponin T, can take weeks to months to normalize or return to baseline values. If troponin levels are not responding, defined as consistently trending down, we recommend proceeding with the initiation of steroid tapering but also recommend considering the initiation of nonsteroidal immunomodulation commensurate with the ESC guidelines.^4^ A caveat with the NCCN guidelines is the difficulty in defining “cardiac function returning to baseline.”^7^ Most patients with ICI myocarditis present with normal left ventricular ejection fraction (LVEF), and for those with depressed LVEF, it is not recommended to wait until LVEF recovers to taper steroids.^2^ Cardiac function returning to baseline could also be interpreted as waiting for arrhythmias to resolve; however, if pulse dose steroids for 3 to 5 days do not result in the resolution of malignant arrhythmias, then additional immunomodulators are suggested as opposed to prolonging high-dose steroids.

## Steroid Duration

The ASCO, SITC, and NCCN guidelines all recommend weaning steroids over 4 to 6 weeks.^5^, ^6^, ^7^ In contrast, the ESC guidelines have specific recommendations for the steroid taper, which results in a much longer taper lasting 17 to 20 weeks (Table 1).^4^ In our practice, we taper steroids over 4 to 6 weeks, and if there is clinical deterioration or a lack of troponin downtrend, we augment treatment with nonsteroidal immunomodulators rather than prolong the steroid duration. Steroids have several side effects including hyperglycemia, infection, muscle weakness confounding overlap muscular irAEs, depression, anxiety, and osteoporosis. The use of antimicrobials for pneumocystis pneumonia prophylaxis while on steroids is suggested. With a rapid steroid taper, the use of vitamin D and calcium supplementation is usually not needed given the lower risk of steroid-induced osteoporosis. Therefore, we recommend the early addition of other immunomodulators as steroid-sparing agents, similar to what is often done in the treatment of non–ICI-related autoimmune diseases. Compared to most autoimmune diseases and studies evaluating steroids in the treatment of viral myocarditis, 4 to 6 weeks is a rapid taper. The shortest steroid tapers evaluated in treating viral myocarditis were 3 months, with the majority lasting 6 to 12 months. The differences highlight the importance of stopping steroids in patients with cancer and allowing them the opportunity to proceed to their next cancer therapy as soon as possible.

## Initiation of Nonsteroidal Immunomodulators

Most current guidelines reserve the use of nonsteroidal immunomodulators for patients who are refractory to steroids alone. The only exception is the ESC guideline, which allows for the use of upfront nonsteroidal immunomodulators in patients presenting with fulminant myocarditis and hemodynamic instability.^4^ This is congruent with clinical practice observations and the literature regarding earlier use of nonsteroidal immunomodulators in addition to steroids. Although up-front use of nonsteroidal immunomodulation is not standard, both the SITC and NCCN guidelines specify that if a patient is not responding to steroids within 24 hours, then nonsteroidal immunomodulators can be considered as adjunct therapy.^6^^,^^7^ The therapeutic options for additional immunomodulation include both broad therapies (plasmapheresis, intravenous immunoglobulin, antithymocyte globulin, and mycophenolate) and specific targeted agents (abatacept [CTLA4-Ig], alemtuzumab [anti-CD52], infliximab [anti–tumor necrosis factor alpha although caution is advised with this agent in the SITC guidelines], and ruxolitinib [Janus kinase inhibitor]).

Currently, abatacept as an adjunct therapy is supported by mechanistic and clinical evidence, which has led to its evaluation in 2 ongoing randomized controlled trials (NCT05335928 and NCT05195645). A recent prospective study evaluated the use of up-front abatacept, ruxolitinib, and lower-dose steroids in 30 patients compared with 10 historical controls who received only up-front higher-dose steroid therapy and found that mortality was much lower at 3% compared to 60%, respectively.^12^ Further studies are needed to evaluate the use of more targeted immunomodulatory agents to allow for lower doses and shorter durations of steroid therapy.

## Conclusions

Corticosteroids are currently the mainstay of treatment for ICI myocarditis, and the use of high-dose steroids has been observed to reduce MACE. The diagnosis and understanding of the severity spectrum of ICI myocarditis are evolving, and ongoing research is evaluating tailored treatments based on clinical presentation. Rapid steroid taper is already recommended by several guidelines, and ongoing trials are evaluating the use of up-front nonsteroidal immunomodulator therapy. As the underlying mechanisms of ICI myocarditis are further delineated, there is a possibility that treatments will move away from steroid-based therapies and toward more targeted immunomodulation, similar to the treatment trends observed in many autoimmune diseases.

## Funding Support and Author Disclosures

Dr Palaskas is supported by the Cancer Prevention and Research Institute of Texas (grant RP200670), National Institutes of Health/National Cancer Institute (grant) 1P01CA261669-01, Andrew Sabin Family Foundation, Replimmune, and Kiniksa Pharmaceuticals. Dr Siddiqui is supported by the American Society of Clinical Oncology-Conquer Cancer Foundation Career Development Award and Prostate Cancer Foundation Young Investigator Award; has received consulting/advisory fees from Merck; and has received research funding to his institution from Regeneron. Dr Deswal is supported in part by the Ting Tsung and Wei Fong Chao Distinguished Chair; and has received consulting fees from Bayer.
